# Frequencies of maternal red blood cell alloantibodies in Port Harcourt, Nigeria

**DOI:** 10.4103/0973-6247.75987

**Published:** 2011-01

**Authors:** Zaccheaus A. Jeremiah, Augustina Mordi, Fiekumo I. Buseri, Teddy. C. Adias

**Affiliations:** *Haematology and Blood Transfusion Science Unit, Department of Medical Laboratory Sciences, College of Health Sciences, Niger Delta University, Wilberforce Island, Bayelsa State, Nigeria*; 1 *Blood Bank Unit, Department of Medical Laboratory Sciences, Braithwaite Memorial Specialist Hospital, Port Harcourt, Nigeria*; 2 *College of Health Technology, Ogbia, Bayelsa State, Nigeria*

**Keywords:** Alloantibodies, frequencies, non-Rh D antibodies, HDFN, Nigeria

## Abstract

**Background:**

Alloantibodies of clinical importance can cause transfusion reactions or hemolytic disease of the fetus and newborn (HDFN). The frequencies of these antibodies have not been reported in our locality.

**Aims:**

To determine the frequency of occurrence of alloantibodies among pregnant women in Port Harcourt, Nigeria.

**Settings and Design:**

This is a prospective study, which was carried out in the Braithwaite Memorial Specialist Hospital, Port Harcourt, Nigeria.

**Materials and Methods:**

Screening and identification of red blood cell alloantibodies was done on the sera of 500 pregnant women using the DiaMed, DiaCell, and DiaPanel reagents (Cressier, Switzerland). ABO and Rh blood groups were done using antisera bought from Biotec (Ipswich, UK).

**Results:**

Alloantibodies were identified in the serum of 17 of the 500 (3.4%) pregnant women. The specificity of the antibodies was as follows: anti-C 6 (1.2%), anti-E 3 (0.6%), anti-Jsb 3 (0.6%), and anti-K 5 (1.0%). No anti-D was identified despite 8.6% of the study population being Rhesus D (Rh D) negative. The distribution of the antibodies was found to be independent of the blood groups of the participants (χ^2^ = 4.050, *P* = 0.670). Blood group O constituted the highest percentage (48.0%).

**Conclusion:**

This study has identified the presence of non-Rh D antibodies to the proportion of 3.4%. Rh D antibody was absent in this population irrespective of the relatively high percentage of Rh D negative women. There is a need to determine the actual risk these antibodies may pose to the antenatal women and to include antibody screening and identification in routine antenatal care.

## Introduction

The red blood cell (RBC) alloantibodies other than naturally occurring anti-A or anti-B are called unexpected RBC alloantibodies and can be found in 0.3%–38% of subjects depending on the group of patients or donor studied and the sensitivity of the test method used.[1,2] Immunization to RBC antigens may result from pregnancy, transfusion, transplantation, or from injection with immunogenic material. In a special care baby unit in Port Harcourt, Nigeria, the overall incidence of neonatal jaundice was 21.4%, 27.8% in outborn and 16.4% in inborn babies.[4] The overall incidence of hemolytic disease of the fetus and newborn (HDFN) varies from place to place ranging from as low as 7.2/10,000 births to as high 14.3/10,000 births.[3–6] Prenatal immunohematologic care of pregnant women requires the investigation of unexpected RBC antibodies in their sera during pregnancy. When RBC antibody screening is positive, it is necessary to determine specificity of the antibody, its clinical importance, and the ability to cross the placenta and cause HDFN. In Port Harcourt, as in other developing parts of the world, type and screen procedure is not routinely carried out as part of the pretransfusion or prenatal test protocol, hence the incidence of maternal RBC alloantibodies and the prevalence of these unexpected antibodies in this locality are not known. With the high incidence rate of neonatal jaundice in Port Harcourt and paucity of information on this subject, it is necessary for a study such as this to be conducted; hence this is the first attempt ever to provide the prevalence of unexpected antibodies in this part of the world.

## Materials and Methods

### Study Area and Population

This study was conducted in Braithwaite Memorial Specialist Hospital, Port Harcourt, and the capital city of Rivers State of Nigeria. The study population consisted of 500 pregnant women recruited from antenatal care unit within a period of 6 months. Their age ranged from 16 to 45 years. The majority of the women were in their second trimesters and multiparous. Their obstetric and transfusion histories were obtained through structured questionnaires, which accompanied antenatal requests.

### Study Design

A prospective cross-sectional design was used in this study. Samples were collected randomly, after obtaining a written or oral informed consent from the participants. Institutional approval was received from the Department of Medical Laboratory Services of the same hospital.

### Laboratory Procedures

### Collection and processing of samples

Two milliliters of whole blood was drawn with syringe (5 mL) through venepuncture using the antecubital vein. One milliliter of whole blood was dispensed into EDTA tube and was used for ABO and Rhesus grouping. The other 1 mL of blood was allowed to clot in a plain bottle, centrifuged at 300 rpm for 5 min, and the serum separated into a separate plain tube with a cap.

### Determination of ABO and Rhesus blood groups

ABO blood grouping was done using anti-A, anti-B, and anti-AB bought from Biotec (Ipswich, UK) with standard tube agglutination technique. All blood group tests were confirmed with known test RBCs. Negative controls were included in all tests. ABO blood group tests were performed only at room temperature. Reverse grouping cells were supplied by the same company, Biotec.

Rhesus grouping was done using anti-D monoclonal reagent bought from Biotec. Rhesus controls were supplied in all tests. Tests were done in tubes and all negative results were confirmed using indirect agglutination test technique with 20% bovine albumin and anti-human globulin (AHG) tests at 37°C. After spinning for 20 s at 100 rpm, the RBC was gently resuspended and immediately observed macroscopically and confirmed microscopically before recording the result as positive or negative.

### Antibody screening and identification

Antibody screening panel (3 cells) and identification panel (11 cells) from DiaMed (Switzerland) was used to screen and identify alloantibodies by tube method in low ionic strength solution, albumin, and AHG phase according to the manufacturer’s instructions.

## Results

The demographic characteristics of the pregnant women are shown in Table 1. A majority of the study population were in the age group of 26–30 years (44.8%) and in their second trimester (60.2%). Thirty-two (6.4%) of them had a history of previous blood transfusion; and 60.4% of the pregnant women were ignorant of their husbands’ Rhesus status. Four (1.7%) reported that their husbands were Rh negative, whereas 95 (37.3%) reported that their husbands were Rh positive. Of the study population, 42.4% have had previous abortion or miscarriage. Twenty-eight (11.2%) responded that they had encountered previous deliveries with neonatal jaundice as shown in Table 2.

**Table 1:** Demographic characteristics of the pregnant women

Characteristics	Number	Percentage
Age group (yr)		
16–20	8	1.6
21–25	60	12.0
26–30	224	44.8
31–35	140	28.0
36–40	50	10.0
41–45	18	3.6
Trimesters		
1^st^	47	9.4
2^nd^	301	60.2
3^rd^	152	30.4
Parity		
Primiparous	142	28.4
Multiparous	258	51.6
Nulliparous	100	20.0

**Table 2:** Previous obstetric and transfusion history

Previous history	Number (%)
Previously transfused?	32 (6.4)
Husbands’ Rh factor known	468 (93.6)
Rh D negative	8 (1.7)
Rh D positive	190 (37.3)
Rh factor unknown	302 (60.4)
**Previous abortion/miscarriage**	
Once	82 (16.4)
Twice	70 (14.0)
Thrice	43 (8.4)
Fourth	12 (2.4)
>4 Times	6 (1.2)
No abortion	300 (60.0)
Previous history of neonatal jaundice	28 (11.2)

Distribution of ABO and Rh blood groups among the 500 pregnant women revealed that group O was 48.0%, A 41.2%, B 7.6%, and AB was 3.2%. Rhesus D (Rh D) positive accounted for 91.4%, whereas Rh D negative was 8.6% [Table 3]. The specificity and prevalence of the 17 clinically significant antibodies detected as shown in Table 4 show anti-C (1.2%) to be most frequent in this study, followed by anti-E and anti-Jsb (0.6%), respectively. The overall frequency of alloantibodies in this population was 3.4%. The antibodies detected were found to occur independent of the participant’s blood groups (χ^2^ = 4.050, *P* = 0.670) as shown in Table 5.

**Table 3:** Distribution of ABO and Rhesus blood groups among the 500 pregnant women

	O	A	B	AB	Total
Rh+	218	187	36	16	457
Rh+%	43.6	37.4	7.2	3.2	91.4
Rh−	22	19	2	0	43
Rh−%	3.3	3.8	0.4	0	8.6
Total	240	206	38	16	500
Total%	48.0	41.2	7.6	3.2	100

**Table 4:** Specificity and percentage of 17 clinically significant non-D antibodies detected in the 500 pregnant women

Antibodies detected	Number (%)
Anti-C	6 (1.2)
Anti-E	3 (0.6)
Anti-Jsb	3 (0.6)
Anti-K	5 (1.0)
Negative	477 (95.4)
Mixed Field*	7 (1.4)
Unidentified**	6 (1.2)
Total	500 (100)

* Mixed field means that the reactions were difficult to interpret

** unidentified antibodies were those antibodies that could not be identified using the DiaPanel cells.

**Table 5:** Distribution of antibody screening results within the blood groups of the study participants

Blood groups	Antibody screening		Total Number (%)
	Positive (%)	Negative (%)	
O Neg	1 (5.9)	21 (4.3)	22 (4.4)
A Pos	5 (29.4)	182 (37.7)	187 (37.4)
AB Pos	0 (0)	16 (3.3)	16 (3.2)
B Neg	0 (0)	2 (0.4)	2 (0.4)
B Pos	1 (5.9)	35 (7.2)	26 (7.2)
A Neg	2 (11.8)	17 (3.5)	19 (3.8)
O Pos	8 (47.1)	210 (43.5)	218 (43.6)
Total	17 (3.4)	483 (96.6)	500 (100)

Pearson Chi-square value 4.050, *P* = 0.670, ns = not significant, Percentage was calculated down the columns.

## Discussion

Irregular RBC antibodies found in the sera of pregnant women have been studied in many parts of the world where prenatal immunohematologic care is given due priority. In this study, the frequency of irregular antibodies in maternal serum was 3.4%. This appears high when compared with values from developed countries, such as Sweden (0.5%), Netherlands (2.7%), and lower when compared with values form other developing countries where higher frequency values of 10.2% in Mexico and 20% anti-D were reported.[3,6–9]

The most frequent and potentially significant non-anti-D antibody in our study was anti-C (1.2%) followed by anti-K (1.0%) then anti-E and anti-Jsb (0.6%), respectively. Anti-C, which was found to be most frequent in this study, corroborates results in other studies where it was found to be most frequent^7^–^10^,^12^ HDFN caused by anti-C is usually mild as the C antigen has weak immunogenicity.[10]

Anti-E can be a naturally occurring IgM antibody, however, IgG anti-E can be found in the sera of pregnant women with a history of previous transfusions and pregnancies. This immune form of anti-E is able to cause a mild to moderate HDFN.[8–11] Anti-K was seen in this study with a frequency of 1.0%. The frequency of K antigen in this locality is not yet known but it is known that after the D antigen, the K antigen is the most immunogenic. HDFN caused by anti-K can be severe.[8] There is evidence that anti-K can recognize K antigens expressed in the early stage of erythroid development in the fetal liver and can cause anemia by suppressing erythropoiesis.[8,12–14]

Jsb has been reported to be common among people of African descent. Anti-Jsb was the least frequent of all the 4 specificities and has been known to be weakly immunogenic. Anti-D was not seen in this study in contrast to 20% anti-D found among pregnant women in Saudi Arabia.[3]

In 2003, Jeremiah and Buseri[15] reported Rhesus antigen and phenotype frequencies in Port Harcourt as follows: D neg (5.0%), C neg (82.3%), c neg (0.2%), E negative (79.5%), and e neg (1.3%). It is therefore not surprising that anti-C and anti-E occurred more frequently in this locality, whereas anti-D was less common. In contrast, the frequency of D-negative has been reported to be approximately 15% among whites and 20%–30% in Middle East and some West African countries. It is not also surprising that variable frequencies of unexpected antibodies were obtained in different regions of the globe. There seemed to be a gradual rise in the anti-D negative population as this study recorded 8.6% as against 5.0% previously reported.

Maternal serum is screened to make sure pregnant mother has no antibodies to react with the fetal cells. HDFN is caused by the mother’s IgG antibodies crossing the placenta and attaching to the baby’s RBCs. It is therefore necessary to know as early as possible in the pregnancy whether HDFN can be a possibility. Determining the specificity of an unexpected alloantibody is important in prenatal testing. If the antibody specificity is known, it is possible to test donor blood for the presence of the corresponding antigen. In prenatal testing, knowledge of the specificity of the antibody helps predict the likelihood of the hemolytic disease of the newborn. Irregular antibodies also cause complications during blood transfusion, thus a reliable crossmatch should include a 2-stage enzyme technique as well as the usual saline and AHG tests. It is emphasized that the antibody screening is an efficient prophylactic measure where complicated crossmatchings are impractical.
